# Generation of an iPSC-derived alveolar rhabdomyosarcoma cell line during directed endothelial differentiation

**DOI:** 10.1016/j.scr.2025.103871

**Published:** 2025-11-12

**Authors:** Randolph K. Larsen, Madeline B. Searcy, Bradley T. Stevens, Katherine E. Gadek, Yang Zhang, Brian J. Abraham, Mark E. Hatley

**Affiliations:** a Department of Oncology, St. Jude Children’s Research Hospital, Memphis, TN, USA; b St. Jude Graduate School of Biomedical Sciences, Memphis, TN, USA; c Department of Computational Biology, St. Jude Children’s Research Hospital, Memphis, TN 38105, USA

**Keywords:** Rhabdomyosarcoma, Endothelial cells, PAX3::FOXO1, Oncofusion, iARMS

## Abstract

Alveolar rhabdomyosarcoma (ARMS) is an aggressive soft tissue sarcoma typically driven by the oncofusion protein PAX3::FOXO1 (P3F). Despite ARMS tumor histology and transcriptome resembling skeletal muscle, these tumors arise in areas devoid of skeletal muscle, indicating that non-myogenic cells can give rise to ARMS. Our lab demonstrated that endothelial progenitors are a cell of origin for rhabdomyosarcoma. Here we provide a protocol for generating iPSC-derived alveolar rhabdomyosarcoma cells (iARMS) during endothelial directed differentiation through enforced expression of P3F. This model allows for dissection of how P3F mediates transformation of endothelial progenitors into aggressive myogenic tumors.

## 1. Resource utility

This protocol was developed to test the capacity of P3F to mediate transformation of endothelial progenitors into aggressive skeletal muscle tumor cells. This method allows for interrogation of the P3F-mediated processes that drive oncogenic transformation during development in a reproducible, scalable, and human system (see Table 1).

## 2. Resource details

**Fig. 1.**
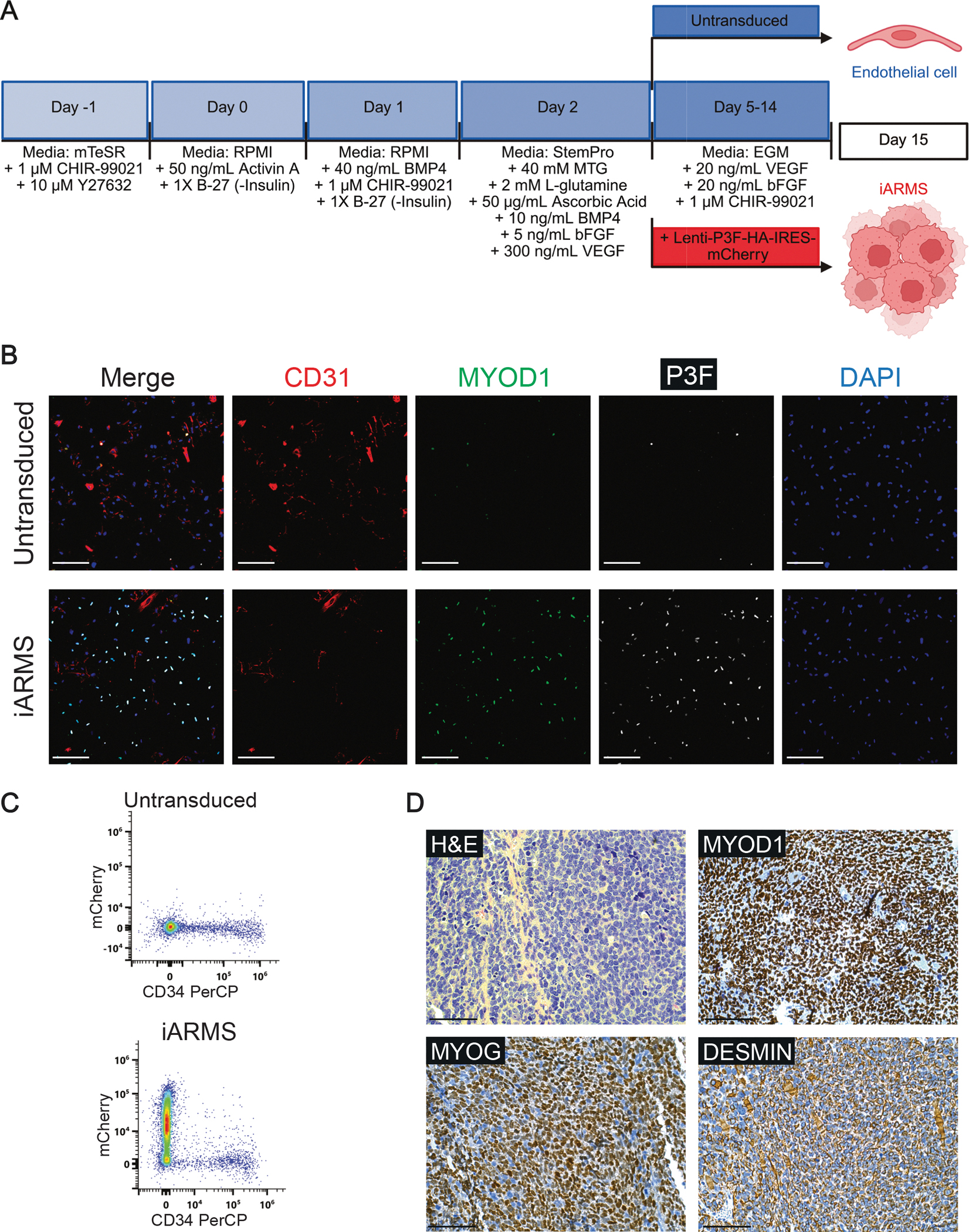
Generation and validation of iPSC-derived alveolar rhabdomyosarcoma (iARMS) cells. (A) Schematic describing the endothelial differentiation protocol ± transduction with Lenti-P3F-HA-IRES-mCherry. (B) Representative immunofluorescent staining for MYOD1 (green), CD31 (red), P3F (white), and DAPI (blue) in iARMS and untransduced cells. Scale bar = 132.2 μm. (C) Representative flow cytometry analysis for mCherry and CD34 in iARMS and untransduced cells. (D) Representative immunohistochemistry images from iARMS tumor xenografts showing H&E and the diagnostic RMS markers MYOD1, MYOG, and DESMIN. Scale bar = 100 μm.

Our laboratory has previously demonstrated that alveolar rhabdomyosarcoma (ARMS) driven by the PAX3::FOXO1 (P3F) oncofusion protein can arise when P3F is expressed in endothelial progenitors in mice (Searcy et al., 2023; Stevens and Hatley, 2025). This model allows for lineage tracing of endothelial progenitors that can be transformed into ARMS and comparison to murine models of ARMS arising from myogenic progenitors (Searcy et al., 2023; Keller et al., 2004). While useful, the murine models are costly, not fully penetrant, and tumors take over 100 days to develop, limiting their tractability for high-throughput screening or studying P3F structure/function. Other groups have developed cell line models of P3F-mediated transformation, but these either require the enforced expression of muscle fate-defining transcription factors or multiple oncogenic drivers to permit P3F-mediated transformation into ARMS (Naini, 2008; , Kalita et al., xxxx). These models provide insight into transformation of muscle progenitors into ARMS, but the artificial expression of myogenic and oncogenic factors may mask functions that P3F independently performs, which can only be revealed through transformation from non-myogenic cells. This protocol models ARMS transformation from a non-myogenic progenitor through the simple addition of P3F during differentiation. To accurately recapitulate the mutational landscape seen in human FP-RMS tumors, we generated *TP53* knockout (*TP53*^*KO*^) human BJFF.6 iPSCs. Detailed methods on the CRISPR-Cas9 generated *TP53*^*KO*^ iPSCs were published previously (Searcy et al., 2023). Briefly, BJFF.6 iPSCs were nucleofected with precomplexed ribonuclear proteins (RNPs) consisting of chemically modified sgRNA, Cas9 protein, and pMaxGFP. GFP+ single cell clones were isolated by FACS and plated on 96-well plates. Knockout clones were identified, expanded, and sequenced confirmed by next generation sequencing analysis. In this protocol, *TP53*^*KO*^ iPSCs are differentiated sequentially to hemogenic mesoderm and then endothelial cells as previously described (Searcy et al., 2023; Palpant et al., 2017). When the hemogenic mesodermal cells are switched into endothelial growth media (EGM) for definitive endothelial differentiation, we transduce them with lentivirus expressing a P3F-HA-IRES-mCherry construct (Fig. 1A). The P3F-expressing cells transform into iPSC-derived alveolar rhabdomyosarcoma cells (iARMS), which lack expression of the endothelial markers CD31 and CD34 and gain expression of the myogenic marker MYOD1 (Fig. 1B–C) (Searcy et al., 2023). Furthermore, iARMS cells grafted into immunocompromised mice form tumors with 100 % penetrance that homogenously resemble human ARMS by immunohistochemistry (Fig. 1D) and gene expression (Searcy et al., 2023). This model provides a scalable human system to study how P3F cooperates with developmental cell state to drive transformation into a muscle tumor and allows for mechanistic dissection of cooperating genetic perturbations (see Table 2).

## Figures and Tables

**Table 1:** Protocol step-by-step procedure.

Procedure	Steps	Sub steps	Comments

**Section A:** Maintenance of *TP53*^*KO*^ iPSCs	1. Prepare Matrigel coated plates by first thawing a 300 μL Matrigel aliquot at 4 °C. Once thawed, dilute the Matrigel aliquot into 30 mL of DMEM/F12 in a 50 mL conical tube on ice and the distribute 1 mL per well into 5, 6-well dishes. Incubate at 37 °C for at least 30 min prior to use. 2. Thaw a vial of*TP53*^KO^ iPSCs by swirling a cryovial of cells in a 37 °C water bath until a small ice crystal remains. Using a 1 mL pipette, transfer the cell solution to 9 mL of iPSC recovery media in a 15 mL conical tube. 3. Centrifuge at 200 × g for 5 min 4. Aspirate supernatant and resuspend cell pellet in 2–3 mL of iPSC recovery media. 5. Aspirate remaining Matrigel solution from one well of a prepared plate and then add resuspended cells to well. 6. Culture cells in incubator at 37 ^0^C, 5 % CO_2_, and 20 % O_2_. 7. After 24 h, replace media with iPSC maintenance media and culture until 80–100 % confluent, replacing with 2 mL of iPSC maintenance media daily. 8. Once cells reach 80–100 % confluency, passage the cells. To passage *TP53*^KO^ iPSCs, aspirate spent media and gently wash with 1 mL of Versene. Aspirate the Versene wash and then add 1 mL fresh Versene to the well and incubate 5–10 min at room temperature. Carefully aspirate the Versene, avoiding lifting the cells. Using 1 mL of iPSC maintenance media, detach the cells from the plate and transfer to a 15 mL conical. Dilute cells with 9 mL of iPSC maintenance media. Aspirate remaining Matrigel solution from a new well and add 1 mL iPSC maintenance media to 1 mL of cell dilution. 9. Continue to change media daily with 2 mL iPSC maintenance media per well. 10. Test cells routinely for mycoplasma contamination using the MycoAlert PLUS Mycoplasma Detection Kit.		1. It is important to keep the Matrigel cold until added to plates. 2. Matrigel should not go through multiple freeze–thaw cycles 3. iPSCs are sensitive to trituration, so minimize pipetting when passaging. 4. We recommend passaging *TP53*^KO^ iPSCs no more than 1:10 resulting in 10 % confluency after passaging. 5. For more detailed passaging instructions and troubleshooting please refer to WiCell Research Institute stem cell protocols.
**Section B:** Reagent resuspensions	1. Activin A – on ice, resuspend pellet in 4 mM HCl in 0.1 % BSA/PBS to a final concentration of 10 μg/mL. Store 250 μL aliquots at −20 ° C for up to 6 months. 2. BMP4 – on ice, resuspend pellet in 4 mM HCl in 0.1 % BSA/PBS to a final concentration of 50 μg/mL. Store 20 μL aliquots at —80 °C for up to 6 months. 3. bFGF – on ice, resuspend pellet in 10 mM Tris (pH 7.6) in 0.1 % BSA/H2O to a final concentration of 50 μg/mL. Store 200 μL aliquots at −20 °C for up to 12 months. 4. VEGF – on ice, resuspend in 0.05 % BSA/H_2_O to a final concentration of 500 μg/mL. Store 20 μL aliquots at −80 °C for up to 12 months. 5. CHIR-99021 – resuspend pellet in sterile DMSO to a final concentration of 25 mM. Store 20 μL aliquots at −20 °C for up to 12 months. 11. Y27632 (ROCKi) – resuspend pellet in sterile DMSO to a final concentration of 10 mM. Store 100 μL aliquots at −20 ° C for up to 12 months.		1. All reagents are resuspended in sterile solutions in a tissue culture hood. No sterile filtering is required.
**Section C:** Media preparations for differentiation	*6. iPSC recovery media* mTeSR Plus + 10 μM ROCKi *7. iPSC maintenance media* mTeSR Plus *8. Day −1 media* mTeSR Plus + 1 μM CHIR-99021 + 10 μM ROCKi *9. Day 0 media* RPMI + 50 ng/mL activin A+ 1X B27 (−1) *10. Day 1 media* RPMI + 40 ng/mL BMP4 + 1 μM CHIR-99021+ 1X B27 (−1) *11. Day 2 media* 1X StemPro with supplement added + 40 mM MTG (1-thioglycerol) + 2 mM L-glutamine + 50 μg/mL ascorbic acid + 10 ng/mL BMP4 + 5 ng/mL bFGF + 300 ng/mL VEGF *12. EGM + single-quots only* EGM with added single-quots *13. Complete EGM* EGM with added single-quots + 20 ng/mL VEGF + 20 ng/mL bFGF + 1 μM CHIR-99021 *14. 4X complete EGM* EGM with added single-quots + 80 ng/mL VEGF + 80 ng/mL bFGF + 4 iM CHIR-99021		1. Make sure factors are diluted in correct buffers, as differentiation will not work if the factors are not correctly resuspended.
**Section D:** Endothelial directed differentiation with PAX3::FOXO1 transduction	1. Prepare a Matrigel coated plate as in Section A, but instead using 500 μL per well of a 24-well dish. 2. When cells are ~ 80 % confluent, follow Section A for lifting the cells with Versene as if they would be passaged. Once in 10 mL, centrifuge cells at 200 × g for 5 min. Aspirate supernatant and gently resuspend cell pellet in 1 mL of Day −1 media. Count cells and then create cell stock required for plating. Recommended plating is 1.6 × 10^5^ cells in 500 μL of Day −1 media per well. Incubate for 24 h at 37 °C. This is designated Day −1. 3. Aspirate spent Day −1 media, gently wash cells with 1 mL of PBS, and then replace with 500 μL per well Day 0 media. Incubate for 17 h at 37 °C. This is designated Day 0. 4. Aspirate spent Day 0 media and replace with 1 mL per well Day 1 media. Incubate 24 h at 37 °C. This is designated Day 1. 5. Aspirate spent Day 1 media and replace with 1 mL per well Day 2 media. Incubate 72 h at 37 °C. This is designated Day 2. 6. On Day 3 of differentiation do not disturb the differentiating cells. Separately, plate 293 T cells to a desired confluency for transfection. Transfect 293Ts with DNA components to make P3F lentivirus. 7. To transfect a 10 cm plate of 293 T cells, begin by adding 60 μL of FUGENE transfection reagent dropwise to 440 μL of serum-free DMEM. Vortex briefly and incubate for 5 min at room temperature. Then, add 2.5 μg of pMD2.G (Addgene #12259), 7.5 μg of psPAX2 (Addgene # 12260) and 10 μg of pSin-P3F-HA-IRES-mCherry dropwise to the transfection solution. Vortex briefly and incubate for 45 min at room temperature. Spin down briefly with a benchtop centrifuge and then add transfection solution dropwise to the plate of 293 T cells. On Day 4 of differentiation do not disturb the differentiating cells. Change media on 293 T cells to EGM +single-quots only. 8. On Day 5 of differentiation, firstly, gelatinize desired number of 6-well dishes by adding 1 mL per well 0.1 % gelatin solution and incubating for a minimum of 30 min at 37 °C. Secondly, collect viral supernatant from 293 T cells and pass through 0.45 μm filter (this is considered 100 % virus solution). Cells will be transduced with a 75 % virus solution. To make the 75 % virus solution combine 2.5 mL of 4X complete EGM for every 7.5 mL of 100 % virus solution. Finally, add ROCKi to a final concentration of 10 μM and polybrene transduction reagent to a final concentration of 8 μg/mL to the 75 % virus solution. Set aside for transduction. 9. To lift Day 5 differentiating cells, aspirate spent media. Then gently wash with 1 mL PBS per well. Add 500 μL of Accutase to each well and incubate at 37 °C for 5 min. Confirm with a microscope that the cells have lifted from the bottom of the plate. Then add 500 μL of EGM + single-quots only to each well and transfer cell solution to a conical tube. Centrifuge cells at 200 × g for 5 min, aspirate the supernatant, and resuspended the cell pellet in 1 mL of complete EGM. Count cells and separate desired number of cells for untransduced controls and for transduction (4.25 × 10^5^ cells for 10 mL of 75 % viral media). Centrifuge cells at 200 × g for 5 min, aspirate the supernatant, and resuspend the cell pellet in either complete EGM + 10 μM ROCKi (untransduced controls) or 75 % virus + 10 μM ROCKi + 8 ig/mL polybrene (transduced cells). 10. Aspirate remaining gelatin solution from plates and dispense cells at a density of 8.5 × 10^4^ cells per well in 2 mL of media in a 6-well dish. Incubate 24 h at 37 °C. 11. Starting on Day 6 of differentiation change media every other day by aspirating spent media and replacing with 2 mL of complete EGM per well. Once the cells reach ~ 80–90 % confluency, passage cells at 1:3 dilution. To passage, aspirate spent media, wash with PBS, and incubate in Accutase for 5 min at 37 °C. Confirm cells have lifted and then add equal volume of complete EGM and transfer to a conical tube. Centrifuge cells at 300 × g for 5 min, aspirate the supernatant, and then resuspend the cell pellet in complete EGM. Before adding to gelatinized culture dish, aspirate remaining gelatin solution. Passage cells onto desired gelatinized plates. 12. Cells are ready for analysis on Day 15 of differentiation.		1. Plating density is not crucial. Successful generation of untransduced endothelial cells and iARMS cells have been generated plating all the way down to half the recommended density. 2. Note that step 3 in particular is only a 17 h incubation. 3. Media changes must happen within 30 min of stated time to ensure proper timing of differentiation. 4. When changing media on cells, work quickly and only a few wells at a time to avoid cells drying out. 5. Note that Day 2 media can be made outside of a sterile tissue culture hood as long as it is sterile filtered inside a sterile tissue culture hood before adding to cells. 6. Transduction of cells can be done without the addition of ROCKi, but viability and success are greatly increased with the addition.
Data analysis	Steps	Sub-steps	Comments
**Section A:** Immunofluorescence for endothelial (CD31) and myogenic (P3F, MYOD1) markers	1. Plate 150,000 cells per well of a gelatinized 24-well dish containing glass coverslips. Ensure glass coverslips are coated in gelatin. For best practice, plate least 2 wells for staining to include a secondary only control. Incubate cells on coverslips least 6 h after plating before beginning immunofluorescence protocol. 2. Wash cells twice for 5 min with ice-cold PBS 3. Fix in 4 % PFA/PBS for 15 min at room temperature. 4. Wash 3 times with PBS for 3 min each and then store at 4 °C in PBS if staining later. If continuing staining, then move to step 5. 5. Permeabilize by incubating slides in 0.1 % Triton X-100/PBS for 15 min at room temperature 6. Wash 3 times with PBS for 3 min each 7. Block in 5 % Normal Donkey Serum (NDS)/0.1 % Triton X-100/PBS for 1 h at room temperature 8. Incubate cells in 300 μL of primary antibody solution diluted in 5 % NDS/0.1 % Triton X-100/PBS overnight at 4 °C a. Primary antibodies include rabbit anti-CD31 diluted 1: 50, mouse anti-MYOD1 diluted 1:50, and rat anti-HA diluted 1:50. b. For secondary only control, incubate in 300 μL 5 % NDS/0.1 % Triton X-100/PBS 9. Wash 3 times with 0.01 % Triton X-100/PBS for 5 min each. 10. Incubate cells in 300 μL of secondary antibody solution diluted in 5 % NDS/0.1 % Triton X-100/PBS for 1 h at room temperature, in darkness. a. Secondary antibodies include Donkey anti-mouse Alexa Fluor 488 1:150, Donkey anti-rabbit Alexa Fluor 568 1:150, Donkey anti-rat Alexa Fluor 647 1:150 b. Recommended dilution is 1:500 for DAPI solution. 11. Wash 3 times with 0.01 % Triton X-100/PBS for 5 min each. 12. Remove coverslip and mount on glass slide in ProLong Diamond Antifade Mountant		
**Section B:** Flow cytometry for transduced cells and endothelial surface markers	1. Begin by lifting cells with Accutase as described in Procedure Section D. Dispense desired number of cells into staining tubes. 2. Filter cell solution through a 70 μm filter into a 50 mL conical. 3. Centrifuge cells at 300 × g for 5 min in a 15 mL conical. Aspirate supernatant and resuspend cell pellet in 50 μL of antibody cocktail diluted in 2 % FBS/PBS. a. Primary antibodies include CD34-PerCP diluted 1:6, CD31-FITC diluted 1:6, VE-CADHERIN-APC diluted 1:6 4. Incubate on ice, in darkness for 30 min. 5. Add 5 mL of 2 % FBS/PBS then centrifuge at 300 × g for 5 min. 6. Aspirate supernatant and resuspend cell pellet in 500 μL of 2 % FBS/PBS to run flow cytometry analysis.		1. Minimum number of cells for analysis is recommended to be 250,000. 2. Untransduced cells are positive control for endothelial cell surface markers. 3. Be sure to include single color controls. 4. iPSCs are notorious for high auto-fluorescence, be sure to use compensation or a spectral cytometer for analysis if possible.
**Section C:** Generating xenograft tumors and immunohistochemistry for RMS markers	1. Begin by lifting cells with Accutase as described in Procedure Section D. 2. Spin down 4,000,000 cells per planned injection, resuspend pellet in 100 μL of growth factor-reduced Matrigel. 3. Inject cells/Matrigel into gastrocnemius of anesthetized immunocompromised mouse. 4. Monitor visually for tumor growth. Tumor burden is typically visible and ready for harvest by 75 days post-engraftment. 5. Fix tumor tissue in 10 % neutral buffered formalin overnight at room temperature and embed in paraffin wax. 6. Heat induced epitope retrieval (HIER) and immunohistochemistry was performed by the St. Jude comparative pathology core. HEIR and staining conditions for each target are listed below: a. DESMIN HIER and staining was performed on Roche Discovery Ultra Autostainer. HIER performed using CC1 buffer for 32 min. Samples incubated in rabbit anti-DESMIN primary antibody 1:500 for 32 min. DESMIN was detected via incubation with OmniMap Rabbit for 16 min. Signal was visualized after 8-minute incubation with ChromoMAP DAB. b. MYOGENIN HIER and staining was performed on Roche Discovery Ultra Autostainer. HIER was performed using CC2 buffer for 32 min. Samples incubated in mouse anti-MYOGENIN primary antibody 1:150 for 60 min. Slides were then incubated in rabbit anti-mouse secondary antibody 1:500 for 16 min. MYOGENIN was detected via incubation with OmniMap Rabbit for 16 min. Signal was visualized after 8-minute incubation with ChromoMAP DAB. c. MYOD1 HIER and staining was performed on Leica BOND MAX Autostainer. HIER was performed using ER2 buffer for 20 min. Samples incubated in rabbit anti-MYOD1 undiluted primary antibody for 15 min. MYOD1 was detected via incubation with Bond Polymer (Included in Refine Detection Kit) for 8 min. Signal was visualized after 10-minute incubation with DAB (Included in Refine Detection Kit).		1. Minimum cell number recommender for successful engraftment is 4,000,000 cells/ injection. 2. Keep syringes/needles on ice prior to filling with cells/matrigel.
**Section D:** RNA-Seq and GSEA	1. Begin by lifting cells with Accutase as described in Procedure Section D. *TERM2GENE:* Our input gene set list combined the gene sets from “h. all.v2023.1.Hs.symbols.gmt” gene sets, as well as the REN_ALVEOLAR_RHADBDOMYOARCOMA_UP signature gene set (https://www.gsea-msigdb.org/gsea/msigdb/human/geneset/REN_ALVEOLAR_RHABDOMYOSARCOMA_UP.html) to align with the study’s focus on particular cellular phenotypes. *eps:* Set to zero to ensure computational stability and prevent any potential division by zero errors. *nPermSimple:* 10,000 permutations *pvalueCutoff:* reporting p value cutoff of 1. 2. Count and spin down 100,000 cells. 3. Resuspend cell pellet in 700 μL of Qiazol Lysis Reagent 4. Add 1.96 μL of 1:100 diluted External RNA Controls Consortium (ERCC) Spike In Mix 1. 5. Extract RNA following Qiagen miRNeasy Micro Kit protocol. 6. Prepare and sequence RNA-seq library following desired method. 7. RNA-seq data was analyzed using the following methods: Raw FASTQ sequences were aligned using HISAT2 (version 2.1.0) (Kim et al., 2015) in paired-end mode using default parameters to version hg38 of the human genome to which the sequences of the ERCC synthetic spike-in RNAs (https://tools.invitrogen.com/downloads/ERCC92.fa) had been added as extra pseudochromosomes. Expression per-gene was quantified using htseq-count (Anders et al., 2015) with parameters “htseq-count – i gene_id –-stranded = reverse – f bam – m intersection-strict,” and version 6 of canonical GRCh38 gene list from RefSeq to which ERCC coordinates and mCherry pseudo-coordinates were added. We used pergene read counts as input for differential expression analysis by DESeq2 (Love et al., 2014). To normalize for differences in sequencing depth and RNA input, size factors were estimated based solely on ERCC spike-in transcripts. Standard DESeq2 processing was applied, including estimation of dispersion and negative binomial modeling, followed by Wald testing to identify differentially expressed genes. 8. For GSEA of bulk RNA-seq: To refine the gene list for GSEAPreranked (Subramanian et al., 2005), only genes in the top half based on their normalized mean expression values across samples (basemean from DESeq2 analysis) were selected. Log2 fold changes (Log2FC) derived from the above DESeq2 analysis were then used to rank retained genes. GSEAPreranked analysis was conducted using the “fgsea” and “cluster-Profiler” packages in R. The analysis utilized the following specific parameters:		
Validations	Steps	Sub-steps	Comments
**Section A: Title**	SEE DATA ANALYSIS		
**Section B: Title**			
**Section C: Title**			
Troubleshooting	Steps	Sub-steps	Comments
**Potential issue A:** Low cell survival on Day 6	Day 5 cells are fragile, so to increase the cell viability and number of cells that make it to Day 6 decrease trituration and make sure that ROCKi is included in both the viral media and the complete EGM used for untransduced cells. Furthermore, cell viability at initial plating and monolayer formation is important for cells making it to Day 5. If cell continue to have low survival on Day 5, we recommend pre-treating for 2 h with ROCKi and then lifting cells for Day −1 with Accutase instead of Versene.		
**Potential issue B:** Low viral transduction	If there is a low number of transduced cells, it may be necessary to determine the optimal range of lentiviral titer by defining the multiplicity of infection that provides high transduction efficiency while minimizing toxicity for your experiment. Additionally, more or less polybrene may help increase the number of transduced cells.		
**Potential issue C:** Low viability of iPSCs during passaging	There are multiple reasons for low viability of iPSCs during passaging. Ensure that Matrigel does not dry out in between aspirating the Matrigel media off the coated plates and plating the iPSCs. Also, make sure that media on iPSCs is changed at least every three days. Changing media frequently ensures that cells remain pluripotent. Passaging cells too frequently can negatively impact cells. If passaging is required more frequently than every three days, try splitting from 1:5–1:12 to allow cells to rest in between passages.		

**Table 2:** Material details.

	Biological Material		
Material name	Manufacturer	Company Cat #	Additional details for use

*TP53* KO BJFF.6 Human iPSCs	Chen and Pruett-Miller (2018) Lee et al. (2015) Searcy et al. (2023)		
HEK 293 T	ATCC	CRL-3216	
	Reagents		
Material name	Manufacturer	Company Cat #	Additional details for use

Growth factor reduced Matrigel basement membrane matrix	Corning	354230	
DMEM/F12	Gibco	11320-033	
mTeSR Plus	Stemcell Technologies	100-0276	
MycoAlert PLUS Mycoplasma Detection Kit	Lonza	LT07-710	
ROCKi (Y27632)	Tocris Bioscience	1254	
Versene	Life Technologies	15040-066	
CHIR-99021	R&D Systems	13122	
RPMI	Life Technologies	11875-093	
Activin A	R&D Systems	338-AC	
B-27 without insulin	Life Technologies	A18956-01	
BMP4	R&D Systems	314-BP	
Stempro-34	Life Technologies	10639-011	
MTG (1-Thioglycerol)	Sigma-Aldrich	M6145-25	
L-glutamine	Life Technologies	25030-081	
L-Ascorbic acid 2-phosphate sesquimagnesium salt hydrate	Sigma-Aldrich	A8960-5G	
bFGF	PeproTech	AF-100-18B	
VEGF	PeproTech	100-20	
EGM BulletKit	Lonza	CC-3124	
pSIN-PAX3::FOXO1-IRES-mCherry	Searcy et al. (2023)		
pSIN-IRES-mCherry	Searcy et al. (2023)		
Polybrene	Sigma-Aldrich	TR-1003	
Porcine gelatin	Sigma	G1890-500G	
PBS	Gibco	10010-023	
Accutase	ThermoFisher Scientific	A1110501	
16 % PFA	Electron Microscopy Science	15,710	
BSA	Millipore Sigma	12659-100GM	
Triton X-100	Bio-Rad	161-0407	
Normal Donkey Serum (NDS)	Millipore Sigma	566460	
FBS	Cytiva HyClone	SH30910.03	
Rabbit anti-CD31	Abcam	ab28364; RRID: AB 726362	Used 1:50 for IF
Mouse anti-MYOD1	Dako	M3512; RRID: AB_2148874	Used 1:50 for IF
Rat anti-HA	Roche	11867423001; RRID:AB_390918	Used 1:50 for IF
Donkey anti-Mouse Alexa Fluor 488	ThermoFisher	A32766; RRID: AB_2762823	Used 1:150 for IF
Donkey anti-Rabbit Alexa Fluor 568	ThermoFisher	A10042; RRID: AB_2534017	Used 1:150 for IF
Donkey anti-Rat Alexa Fluor 647	ThermoFisher	A48272; RRID: AB_2893138	Used 1:150
Anti-CD34-PerCP	BD Biosciences	340430; RRID: AB_400034	Used 1:6 for Flow analysis
Anti-CD31-FITC	BD Biosciences	555445; RRID: AB_395838	Used 1:6 for Flow analysis
Anti-VE-Cadherin-APC	Invitrogen	17-1449-42; RRID:AB_10804754	Used 1:6 for Flow analysis
ProLong Diamond Antifade Mountant	Invitrogen	P36965	
Scigen VIP Fixative, 10 % Neutral Buffered Formalin	Fisher Scientific	23-730-586	
CC1 Buffer	Roche	950-500	
CC2 Buffer	Roche	950-107	
ER2	Leica	AR9640	
Rabbit anti-DESMIN	ThermoFisher	RB-9014; RRID: AB_149769	
Mouse anti-MYOGENIN	Abcam	Ab1835; RRID: AB_302633	
Rabbit anti-mouse	Abcam	Ab133469; RRID: AB_2910607	
Rabbit anti-MYOD1	Cell Marque	386R-18	
OmniMap Rabbit	Roche	760-4311	
Refine Detection Kit	Leica	DS9800	
ChromoMap DAB	Roche	760-159	
Qiazol	Qiagen	79306	
Qiagen miRNeasy Micro Kit	Qiagen	217084	
ERCC RNA Spike-In Mix	Invitrogen	4456740	
	Solutions		
Solution name	Components	Concentration and quantity	Additional details for use

iPSC recovery media	mTeSR Plus ROCKi	10 μM	
iPSC maintenance media	mTeSR Plus		
Day −1 media	mTeSR Plus		
	CHIR-99021	1 μM	
	ROCKi	10 μM	
Day 0 media	RPMI		
	Activin A	50 ng/mL	
	B27 (−1)	1X	
Day 1 media	RPMI		
	BMP4	40 ng/mL	
	CHIR-99021	1 μM	
	B27 (−I)	1X	
Day 2 media	1X StemPro w/supp.		
	MTG (1-thioglycerol)	40 μM	
	L-glutamine	2 mM	
	Ascorbic acid	50 μg/mL	
	BMP4	10 ng/mL	
	bFGF	5 ng/mL	
	VEGF	300 ng/mL	
EGM + single-quots only	EGM bullet kit with the single-quots added		
Complete EGM	EGM bullet kit with the single-quots added	20 ng/mL	
	VEGF	20 ng/mL	
	bFGF	1 μM	
	CHIR-99021		
4X complete EGM	EGM bullet kit with the single-quots added	80 ng/mL	
	VEGF	80 ng/mL	
	bFGF	4 μM	
	CHIR-99021		
Permeabilization	PBS	0.1 %	
solution	Triton X-100		
Blocking solution	PBS		
	Triton X-100	0.1 %	
	NDS	5 %	
	Equipment		
Equipment name	Manufacturer	Company Cat #	Additional details for use

Sterile biological	ThermoFisher	1323TS	
safety cabinet (1300 Series A2)	Scientific		
Humidified tissue culture incubator (HERACell VIOS 250i)	ThermoFisher Scientific	51033782	
Centrifuge	ThermoFisher Scientific	75009525	
Cell counter (Countess II)	Invitrogen	AMQAX1000	
DMi8 Thunder	Leica		Instant
Imager inverted fluorescent microscope			Computational Clearing performed on all images shown in Fig. 1B
Aurora Spectral Analyzer	Cytek Biosciences	Aurora	
Roche Discovery Ultra Autostainer	Roche	05987750001	
Leica BOND MAX Autostainer	Leica	49.0051	
	Laboratory supplies		
Product name	Manufacturer	Company Cat #	Additional details for use

Conical tubes (15 and 50 mL)	Thermo Scientific	339651 and 339653	
6-well dish	Corning (Fisher Scientific)	720083	
Sterile 0.45 μm filter	Thermo Scientific	725-2545	
24-well dish	Corning	353047	
10-cm plate	TPP	93100	
Sterile 70 μm filter	Falcon	352350	
Serological pipettes (5, 10, 25 and 50 mL)	Costar	4487, 4488, 4489, and 4490	
25 G sterile needles	BD Biosciences	305125	
1 mL syringe	BD Biosciences	309659	
	Software and Datasets		
Name	Website or location	If commercial, Company Cat # and version	Additional details for use

FlowJo	https://www.flowjo.com/solutions/flowjo	Version 10.8.1	

**Resource table:** 

Unique stem cell line identifier	BJFF.6 (RRID: CVCL_VU02) (Chen and Pruett-Miller, 2018) (Lee et al., 2015)
Contact information of distributor	Mark Hatley (mark.hatley@stjude.org)
Type of cell line and species on which the protocol was tested Type of cell line and species to which the protocol can be applied	Human BJFF.6 *TP53*^*KO*^ iPSCs
Protocol type	Genetic modification Lentiviral insertion Directed endothelial differentiation Growth factor exposure Transformation validation Immunocytochemistry Flow cytometry Immunohistochemistry
Genetic Modification	Lentiviral transduction using pSIN-P3F-HA-IRES-mCherry vector (Searcy, 2023)
Relevant disease/developmental process	Endothelial Development, Rhabdomyosarcoma Development
Gene/locus	N/A
Requires specialized equipment	N/A
Ethical approval	Committee Name: St. Jude Clinical Trials and Scientific Review Committee (CT-SRC) Protocol Name: HatleyiPSC2020 (Approved 11/9/2020)

## Data Availability

Data will be made available on request.
